# Long-term outcome in medical patients aged 80 or over following admission to an intensive care unit

**DOI:** 10.1186/cc9984

**Published:** 2011-01-24

**Authors:** Antoine Roch, Sandrine Wiramus, Vanessa Pauly, Jean-Marie Forel, Christophe Guervilly, Marc Gainnier, Laurent Papazian

**Affiliations:** 1 Medical Intensive Care Unit, Hôpital Nord, Chemin des Bourrely, Marseille, 13015, France; 2 Department of Medical Information, Hôpital Sainte Marguerite, 269 Boulevard de Sainte Marguerite, Marseille, 13274, France

## Abstract

**Introduction:**

The aim of this study was to evaluate factors influencing short- and long-term survival in medical patients aged 80 and over following admission to an intensive care unit.

**Methods:**

All patients aged 80 years or over and admitted between 2001 and 2006 were included in this study. Survival was evaluated between the time of admission and June 2009; factors associated with mortality were determined. Health-related quality of life was evaluated using Short Form (SF)-36 in long-term survivors.

**Results:**

For the 299 patients included (mean age, 84 ± 4 y), hospital mortality was 55%. Factors independently associated with hospital mortality were a higher SAPS II score at ICU admission; the existence of a fatal disease as reflected by the McCabe score and a cardiac diagnosis at admission. In the 133 hospital survivors, median survival time was 710 days (95% CI, 499-921). Two-year mortality rates were 79% of the initial cohort and 53% of hospital survivors. The standardized ratio of mortality at 2 years after hospital discharge was 2.56 (95% CI, 2.08-3.12) when compared with age- and gender-adjusted mortality of the general population. Factors independently associated with mortality at 2 years after hospital discharge were SAPS II score at ICU admission and the McCabe score. Conversely, functional status prior to admission as assessed by Knaus or Karnofsky scores was not associated with long-term mortality. In long-term survivors, SF-36 physical function scores were poor but scores for pain, emotional well-being and social function were not much affected.

**Conclusions:**

The severity of acute disease at admission influences mortality at the hospital and following discharge in patients aged 80 or over. Although up to 50% of patients discharged from the hospital were still alive at 2 years, mortality was increased when compared with the general population. Physical function of long-term hospital survivors was greatly altered.

## Introduction

As in many countries, in France, average age and life expectancy of the population are increasing [1]. Because of this, a growing number of much older patients are being admitted to the intensive care unit (ICU). There is some evidence to suggest that age is a restrictive factor for ICU admission [2,3] and that it determines treatment intensity [4,5]. However, even though an increased risk of mortality accompanies old age [6-10], most studies suggest that age alone does not represent a strong predictor for mortality [4]. However, few data concerning long-term survival after ICU admission in much older medical patients are currently available. Since these may be the patients with the worst prognosis at the hospital and following discharge [11], a better knowledge of factors associated with long-term outcome in this population is warranted.

The goal of the present study was to evaluate short- and long-term survival in a large cohort of medical patients who were at least 80 years of age. Moreover, health-related quality of life (HRQOL) was prospectively evaluated in long-term survivors by means of the Short Form-36 (SF-36) questionnaire [12].

## Materials and methods

The protocol was approved by the ethics committee of the Institut Fédératif de Recherche 48 de la Faculté de Médecine de Marseille (Marseille, France), which, in accordance with French legislation, waived the need for informed consent of patients whose data were retrospectively studied. In regard to phone interviews, participants themselves or a close family member gave informed consent to participation in the study.

This study was performed in the Hôpital Sainte-Marguerite, an adult acute, tertiary care university teaching hospital. Our ICU is a 12-bed medical unit admitting 400 adult patients per year for a mean stay of 9 days. Patients were admitted after an evaluation by an intensivist. We had no specific admission criteria. Before ICU admission, we tried to obtain information regarding prehospital disability, presence of any underlying disease, number of organ failures, and patient wishes. In the absence of this information, the patient was nevertheless admitted. All patients who were at least 80 years of age and who were admitted to our ICU for medical reasons between January 2001 and December 2006 were retrospectively included in this study. Only the first stay of patients who were admitted several times during the study period was included in the study. Vital status was determined in June 2009 from the patient's record or by calling the primary care physician or proxies. The following data were prospectively collected for each patient while he or she was present at the ICU: gender, severity of illness at admission according to the Simplified Acute Physiologic Score II (SAPS II) [13] and the Sequential Organ Failure Assessment (SOFA) score [14], duration of ICU stay, initiation of mechanical ventilation or renal replacement therapy, treatment limitation during ICU stay (defined as the decision not to use mechanical ventilation or renal replacement therapy or both), occurrence of ICU-acquired pneumonia according to predefined criteria [15], and ICU and hospital mortality. The reasons for ICU admission were classified into the following subgroups: respiratory disease, cardiac disease, sepsis, renal disease, coma or neurological disease, digestive diseases, or other reasons. The severity of any underlying disease present at the time of ICU admission was classified according to the McCabe score [16]. This classification uses precise criteria to group patients according to disease fatality: no fatal disease, ultimately fatal disease (expected to be fatal in the next 5 years), or rapidly fatal disease (expected to be fatal in the next year). Functional status before admission was routinely assessed by means of Karnofsky [17] and Knaus [18] scores. Shortly after patient admission, the physician in charge prospectively documented these scores on the patient's computerized record on the basis of information collected from the patient, proxies, and other physicians. The time point for the determination of functional status was just before the current hospital admission.

### Long-term follow-up and health-related quality of life measurement

We used the SF-36 questionnaire [12] to describe HRQOL. Each heading in the questionnaire is represented by one or more items with scores ranging from 0 to 100, 0 being the worst score. SF-36 questionnaires were completed during phone interviews that were all performed by the same investigator in June 2009. After information on vital status was collected from the primary care physician, patients or their close family members were called. Participants themselves or a close family member gave informed consent to participation in the study. Patients were interviewed directly, but assistance from a family member was allowed.

### Analysis

Descriptive statistics included frequency analysis (percentages) for nominal variables and means ± standard deviations (SDs) or medians and interquartile ranges (IQRs) for continuous variables, according to their distribution. The survival curve after hospital discharge and median survival time were estimated by the Kaplan-Meier method, and patients who were still alive at the date of follow-up (15 June 2009) were censored. The survival of our cohort was compared with the survival curve for the French population as a whole, established from mortality data obtained in 42,336 people who had a mean age of 84 years [19]. The standardized mortality ratio (SMR) method was used to compare the hospital mortality observed in our cohort with SAPS II-predicted mortality and to compare the mortality observed in our cohort at 2 years after discharge with age- and gender-adjusted mortality of the general population. We used a Cox survival analysis to identify independent predictors of mortality at the hospital and of mortality at 2 years after hospital discharge. For the latter, survival was measured from the first day after discharge, and patients alive at 2 years were censored. First, univariate analysis was performed for each potential factor. Factors with a *P *value of less than 0.2 in the univariate analysis were then introduced as part of a backward stepwise Cox proportional hazard model. Hazard ratios and 95% confidence intervals (CIs) were calculated. In the final multivariate model, a *P *value of less than 0.05 was considered significant. Factors significantly associated with mortality in the multivariate model were tested for a possible interaction. Statistical analysis was performed by means of SPSS 15.0 software (SPSS, Inc., Chicago, IL, USA).

## Results

### Patients

Of the 2,411 patients admitted to the ICU during the 6-year study period, 299 (12.4%) who were at least 80 years old (84 ± 4 years; range of 80 to 97) were included (Table 1). Among them, 176 (59%) were mechanically ventilated for a median duration (IQR) of 4 days (2 to 9). The median duration (IQR) of ICU stay was 5 days (3 to 9). Eleven patients had one or more ICU readmissions, but none of them had been discharged from the hospital between ICU stays.

**Table 1:** Population characteristics and factors associated with hospital mortality

	All patients	Hospital survivors	Hospital non-survivors	HR univariate [95% CI]	*P *univariate	Adjusted HR [95% CI]	*P *multivariate
Number	299	133	166				
Age in years, mean ± SD	84 ± 4	84 ± 4	84 ± 4	1.02 [0.98; 1.06]	0.35		
Males	140 (47)	58 (44)	82 (49)	0.90 [0.66; 1.22]	0.51		
Mechanically ventilated	176 (59)	48 (36)	128 (77)	2.38 [1.65; 3.43]	<0.001		
Renal replacement therapy	21 (7)	2 (1)	19 (11)	1.54 [0.95; 2.49]	0.08		
SAPS II, mean ± SD	52 ± 22	42 ± 13	61 ± 24	1.04 [1.03;1.05]	<0.001	1.03 [1.03; 1.04]	<0.001
SOFA score, mean ± SD^a^	7 ± 4	4 ± 3	8 ± 4	1.18 [1.14; 1.23]	<0.001		
McCabe score					<0.001		<0.001
No fatal disease	129 (43)	69 (52)	60 (36)	1			
Fatal disease at 5 years	117 (39)	53 (40)	64 (39)	1.28 [0.90; 1.83]	0.17	1.40 [0.97; 2.02]	0.07^b^
Fatal disease at 1 year	53 (18)	11 (8)	42 (25)	2.66 [1.78; 3.96]	<0.001	3.17 [2.08; 4.83]	<0.001^b^
Knaus score					0.04		
No limitation	45 (15)	27 (20)	18 (11)	1			
Slight limitation	121 (41)	53 (40)	68 (41)	1.51 [0.89; 2.54]	0.12		
Severe limitation	103 (34)	42 (32)	61 (37)	1.61 [0.95; 2.73]	0.08		
Bedridden	30 (10)	11 (8)	19 (11)	2.55 [1.33; 4.87]	0.004		
Karnofsky score, median (IQR)	80 (50-90)	80 (60-90)	80 (50-90)	0.99 [0.98; 0.99]	0.03		
Nosocomial pneumonia	30 (10)	7 (5)	23 (14)	0.77 [0.49; 1.22]	0.27		
Treatment limitation^c^	69 (23)	25 (19)	44 (26)	1.38 [0.80-2.05]	0.15		
Admission diagnosis					<0.001		0.009
Respiratory	141 (47)	71 (53)	70 (42)	0.77 [0.51; 1.16]	0.21	0.88 [0.57; 1.34]	0.54
Coma or neurological	56 (19)	22 (17)	34 (20)	1.23 [0.77; 1.98]	0.39	0.97 [0.59; 1.58]	0.91
Cardiac	43 (14)	10 (8)	33 (20)	3.04 [1.87; 4.9]	<0.001	2.28 [1.38; 3.77]	<0.001
Sepsis	29 (10)	12 (9)	17 (10)	1.48 [0.83; 2.6]	0.19	1.32 [0.74; 2.38]	0.35
Digestive	17 (6)	11 (8)	6 (4)	0.38 [0.16; 0.90]	0.029	0.56 [0.23; 1.35]	0.19
Renal	7 (2)	3 (2)	4 (2)	0.74 [0.26; 2.12]	0.58	0.46 [0.16; 1.33]	0.15
Other	6 (2)	4 (2)	2 (1)	0.83 [0.20; 3.39]	0.8	1.51 [0.37; 6.24]	0.57

Data are presented as number (percentage) unless otherwise specified. All variables with a *P *value of less than 0.2 after univariate analysis were introduced in the multivariate analysis. ^a^Simplified Acute Physiology Score (SAPS II) but not Sequential Organ Failure Assessment (SOFA) score was introduced in the multivariate analysis; ^b^versus no fatal disease; ^c^decision not to use mechanical ventilation or renal replacement therapy or both. CI, confidence interval; HR, hazard ratio; IQR, interquartile range; SD, standard deviation.

### Intensive care unit and hospital mortality

ICU mortality was 46% (138/299), and mortality throughout the duration of hospital stay was 55% (166/299). Factors associated with hospital mortality are detailed in Table 1. After multivariate analysis, the factors found to be significantly associated with increased hospital mortality were a higher SAPS II at ICU admission, the existence of a fatal disease as reflected by the McCabe score, and a cardiac diagnosis at admission. No significant interaction between factors associated with hospital mortality was found. The SMR of our cohort was 0.99 (95% CI 0.84 to 1.18) when compared with SAPS II-predicted mortality.

### Mortality at 2 years after hospital discharge

**Figure 1:**
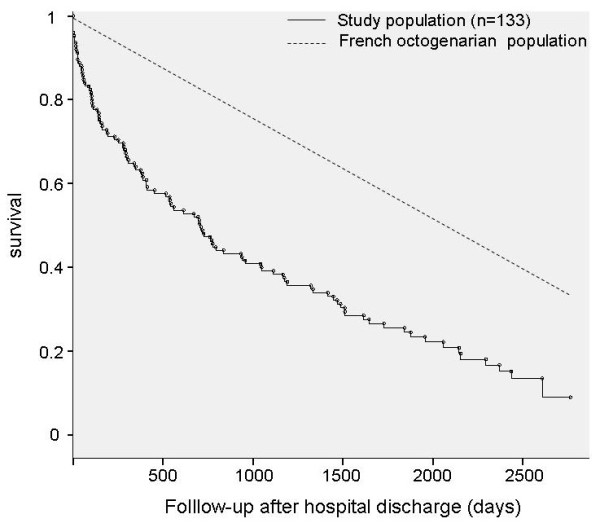
**Kaplan-Meier survival curve of hospital survivors in comparison with that of the general French population**. Age in both groups was a mean of 84 years. Mortality data for the latter were obtained from [19].

Of the 133 patients (45% of the initial cohort) who were discharged from the hospital, 49 died over the course of the first year after discharge and 21 died during the second year (no loss to follow-up). Thus, 1-year mortality after admission was 72% (215/299) and 2-year mortality after admission was 79% (236/299). Two-year mortality in hospital survivors was 53%, whereas in the same age group for the general French population, it was 18% [19]. Age- and gender-adjusted SMR of our cohort was 2.56 (95% CI 2.08 to 3.12) when compared with the general population. The survival curve after hospital discharge is shown in Figure 1. The estimated median survival time after hospital discharge was 710 days (95% CI 499 to 921). We analyzed which factors, available at ICU admission, could be predictive of mortality at 2 years after hospital discharge (Table 2). After multivariate analysis, the factors found to be significantly associated with increased mortality were a higher SAPS II at ICU admission and the existence of a fatal disease as reflected by the McCabe score. Conversely, functional status, as evaluated by the Knaus classification or the Karnofsky index before ICU admission, was not significantly associated with mortality at 2 years in hospital survivors. No significant interaction between factors associated with mortality at 2 years after hospital discharge was found. When multivariate analysis was conducted in patients who were still alive 30 days (*n *= 120), 90 days (*n *= 112), or 180 days (*n *= 100) after discharge, SAPS II was also significantly associated with mortality at 2 years after hospital discharge. However, it was no longer associated with mortality (*P *= 0.13) in survivors at 1 year after discharge (*n *= 88).

**Table 2:** Characteristics of hospital survivors and factors associated with mortality at 2 years

	Hospital survivors	Two-year survivors	Two-year non-survivors	HR univariate [95% CI]	*P *univariate	Adjusted HR [95% CI]	*P *multivariate
Number	133	63	70				
Age in years, mean ± SD	84 ± 4	84 ± 4	84 ± 3	1.00 [0.94; 1.08]	0.84		
Males	58 (44)	31(49)	27 (39)	1.37 [0.84; 2.23]	0.21		
Mechanically ventilated	48 (36)	27 (42)	21 (30)	1.53 [0.93; 2.50]	0.09		
Renal replacement therapy	2 (1)	1 (1)	1 (1)	0.72 [0.09; 5.20]	0.75		
SAPS II, mean ± SD	42 ± 13	40 ± 13	44 ± 12	1.02 [0.99; 1.03]	0.06	1.02 [1.00; 1.04]	0.03
SOFA score, mean ± SD	4 ± 3	4 ± 3	4 ± 3	1.02 [0.94; 1.11]	0.6		
McCabe score					0.029		0.018
No fatal disease	69 (52)	39 (62)	30 (43)	1		1	
Fatal disease at 5 years	53 (40)	22 (35)	31 (44)	1.62 [0.96; 2.73]	0.07	1.81 [1.06; 3.13]	0.03
Fatal disease at 1 year	11 (8)	1 (2)	10 (14)	2.60 [1.22; 5.54]	0.013	2.62 [1.23; 5.59]	0.013
Knaus score					0.38		
No limitation	27 (20)	9 (14)	18 (26)	1			
Slight limitation	53 (40)	26 (41)	27 (39)	1.54 [0.72; 3.30]	0.26		
Severe limitation	42 (32)	23 (36)	21 (30)	1.86 [0.86; 4.01]	0.11		
Bedridden	11 (8)	6 (10)	5 (7)	2.13 [0.76; 6.00]	0.15		
Karnofsky score, category	80 (60-90)	80 (50-90)	80 (50-90)	0.99 [0.98; 1.00]	0.35		
Nosocomial pneumonia	7 (5)	3 (5)	4 (6)	0.75 [0.24; 2.39]	0.63		
Admission diagnosis					0.43		

Data are presented as number (percentage) unless otherwise specified. All variables with a *P *value of less than 0.2 after univariate analysis were introduced in the multivariate analysis. ^a^Versus no fatal disease. CI, confidence interval; HR, hazard ratio; SAPS II, Simplified Acute Physiology Score II; SD, standard deviation; SOFA, Sequential Organ Failure Assessment.

### Long-term health-related quality of life

**Figure 2:**
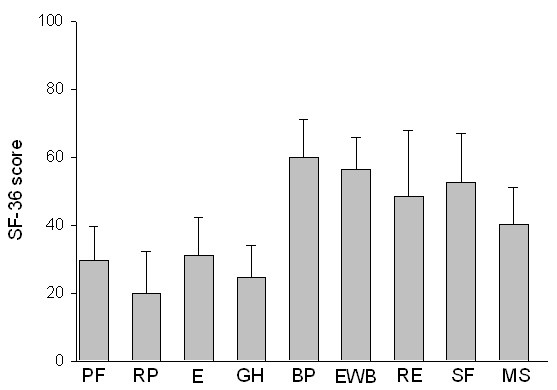
**Short Form-36 (SF-36) scores in 24 prospectively evaluated long-term survivors**. Scores are presented as mean ± standard deviation. BP, bodily pain; E, energy; EWB, emotional well-being; GH, general health; MS, mean score; PF, physical function; RE, emotional role; RP, physical role; SF, social function.

HRQOL using SF-36 was prospectively evaluated in the 24 patients who were still alive at the time of evaluation in June 2009 (no loss to follow-up). Their median age (IQR) at evaluation was 89 years (87 to 92). The median time (IQR) between hospital discharge and SF-36 evaluation was 63 months (56 to 85). Twenty-one patients answered the questionnaire by themselves, and 3 with the help of a third party. Scores of physical function were low (Figure 2). Indeed, mean scores ± SD were 29 ± 12 for physical function, 20 ± 12 for physical role (which evaluates limitations due to physical function), 31 ± 11 for energy, and 24 ± 10 for general health (which evaluates the perception of health). In contrast, scores of bodily pain (56 ± 10), emotional well-being (56 ± 9), social function (52 ± 15), and emotional role (48 ± 22) (which evaluates activity limitations due to mental health) were not much affected.

## Discussion

This follow-up study was conducted in a population of severely ill medical patients who were at 80 years old (84 ± 4 years) and who were admitted to the ICU. In this population, hospital mortality was 55%, and 47% of hospital survivors were alive at 2 years. Both hospital and post-discharge mortality rates were dependent mainly on the severity of acute illness and on the existence of a pre-existent underlying disease. Conversely, pre-admission functional scores as evaluated by the Knaus classification or the Karnofsky index before ICU admission did not affect mortality at the hospital or over the 2-year period following discharge.

The reported hospital mortality rate of 55% in our patients is higher than in several recent studies of much older patients, in which hospital mortality rates ranging from 12% to 41% were reported [5,20-26]. However, some of these studies were performed in patients who were just 65 years old or older [20-25], in populations with lower severity scores or lower rates of mechanical ventilation [5,20,25,26], in surgical or mixed populations of medical and surgical patients [20-22], or in patients with a previously healthy status [23]. Conversely, De Rooij and colleagues [11] reported a 56% mortality rate in 146 medical patients who were at least 80 years old, with a rate of mechanical ventilation and severity scores that were similar to those described in the present study. Upon comparison, medical patients had a worse prognosis than surgical patients. Subsequently, these authors reported a 75% mortality rate at 2 years after admission [11], which is close to our results.

The long-term follow-up indicates that mortality of our patients in the 2 years after discharge was two- to three-fold the mortality of the general French population of the same age. However, after this time, the evolution of survival over time was comparable to that of the general population. Therefore, we analyzed which factors could be associated with prognosis during this period of over-mortality. We observed that severity score at the time of admission independently affected mortality in this 2-year period following discharge. This is an interesting result since it shows that the severity of an acute illness will influence outcome after ICU and hospital discharge. The results of additional analysis in survivors at different time points after discharge suggest that the severity at admission negatively influences prognosis mainly during the first months after discharge. Conversely, we found that Knaus and Karnofsky scores of functional status before admission did not influence mortality in the 2 years after discharge. In a previous report, Bo and colleagues [25] showed that dependence for regular daily activities was independently associated with hospital mortality in medical ICU patients who were at least 65 years old. In that study, severity scores and hospital mortality (14.7%) were much lower than in our patients, far fewer patients required mechanical ventilation, and two thirds were independent for regular daily activities before ICU admission. Similarly, Sacanella and colleagues [23] found that full autonomy before ICU admission was independently associated with a lower mortality rate after discharge in patients at least 65 years old. In contrast, in older patients such as those of the present study, only 15% of patients had no functional limitation. Therefore, the ability to identify functional status as a prognostic factor in such a homogeneous population is limited. However, since functional limitation is frequent in much older patients, our results suggest that care should be taken when using it to make admission decisions and in the determination of treatment intensity in this category of patients. Nevertheless, our results contrast with those of Boumendil and colleagues [24], who found a severe or total functional limitation to be independently associated with mortality after discharge in 233 medical patients who were at least 80 years old. In this latter study, included patients had lower severity scores and a much lower ICU mortality rate (16.3%) in comparison with those of the present study. Discrepancies between previous studies and the present report on the influence of functional status on long-term mortality could be partly explained by a higher rate of patients with severe limitation in our study and by the selection in other studies of patients in good condition, who are able to recover well after ICU discharge.

We prospectively evaluated HRQOL in the 24 long-term survivors. The scores for physical function were poor, but scores for bodily pain, emotional well-being, and social function were not much different from those of other populations of octogenarians [27]. These latter results could be positively interpreted. Indeed, Nilsson and colleagues [28] interviewed healthy individuals who were 77 to 87 years old on the quality of their lives and showed that the importance of material values declined but that the importance of social relations and spending time by oneself increased with increasing age, suggesting that 'quality of life' has a different meaning for older individuals than it does for younger ones. In ICU patients, Tabah and colleagues [21] recently found that quality of life was similar between (a) patients who were at least 80 years old and who survived 1 year after discharge and (b) reference populations of the same age and that quality of life was not modified after the ICU stay. In contrast, in a previous study in a similar population, the same group showed a decrease in quality of life 1 year after ICU stay [2]. In the present study, we evaluated HRQOL of long-term survivors a median of 5 years after discharge. Recently, Cuthbertson and colleagues [29] showed that the physical component of quality of life worsened faster in the 5 years following ICU stay than in the general population. Additionally, Unroe and colleagues [30] showed that age was associated with an increased risk of high functional dependency following prolonged mechanical ventilation. Therefore, our results of high long-term mortality in the most severely ill medical patients in the age group discussed here and of severe functional disability in long-term survivors could help physicians to explicitly discuss treatment decisions with surrogates on the basis of the future functional dependence that patients will likely experience.

This study has several limitations. First, this is a single-center study, and owing to variations in admission policies, caution must be taken in translating these results to other ICUs. Second, the analysis of factors associated with survival after ICU discharge did not include parameters occurring after ICU discharge, such as repeated ICU admissions or institutionalization. However, the goal of this study was to help clinician decision-making on the basis of data available during ICU stay. Third, only very few patients were long-term survivors, and further studies are required to evaluate HRQOL in much older patients in the years following ICU discharge. Finally, although we found that functional status prior to ICU admission was not associated with mortality either at the hospital or after discharge, it was determined after admission by physicians using information obtained from the patient or from proxies. Moreover, we cannot rule out that other scores of functional status may be more accurate in predicting long-term outcome in much older patients.

## Conclusions

Our study provides information about short- and long-term outcome for a large group of much older patients in the medical ICU. We showed that the severity of acute disease at admission influences mortality at the hospital and also after discharge. Conversely, functional status prior to admission did not influence short- and long-term prognosis in this category of frequently dependent patients. Although up to 50% of patients discharged from the hospital were still alive at 2 years, mortality in the 2 years following discharge was three times the mortality observed in the same age group in the general population. Finally, physical function of long-term hospital survivors was greatly altered, but other components of HRQOL were not much affected when compared with the general population. These results could help the clinician make decisions with regard to the most severely ill patients in this age group.

## Key messages

• Severity of acute disease at admission is associated with mortality at the hospital and also after discharge in much older patients in the medical intensive care unit.

• Mortality for much older patients in the 2 years after discharge is three times the mortality observed in the same age group in the general population.

• The physical component of health-related quality of life is greatly altered in long-term survivors.

## Abbreviations

CI: confidence interval; HRQOL: health-related quality of life; ICU: intensive care unit; IQR: interquartile range; SAPS II: Simplified Acute Physiologic Score II; SD: standard deviation; SF-36, Short Form-36; SMR: standardized mortality ratio.

## Competing interests

The authors declare that they have no competing interests.

## Authors' contributions

AR conceived the study, participated in its coordination, and drafted the manuscript. SW, JMF, CG, and MG collected data and helped to draft the manuscript. VP participated in the design of the study and performed the statistical analysis. LP participated in the design of the study and helped to draft the manuscript. All authors read and approved the final manuscript.
